# *QuickStats:* Percentage* of Women Aged 22–44 Years Who Have Ever Cohabited with an Opposite-Sex Partner,^†^ by Education^§^ — National Survey of Family Growth, United States, 2006–2010 and 2015–2019

**DOI:** 10.15585/mmwr.mm7002a5

**Published:** 2021-01-15

**Figure:**
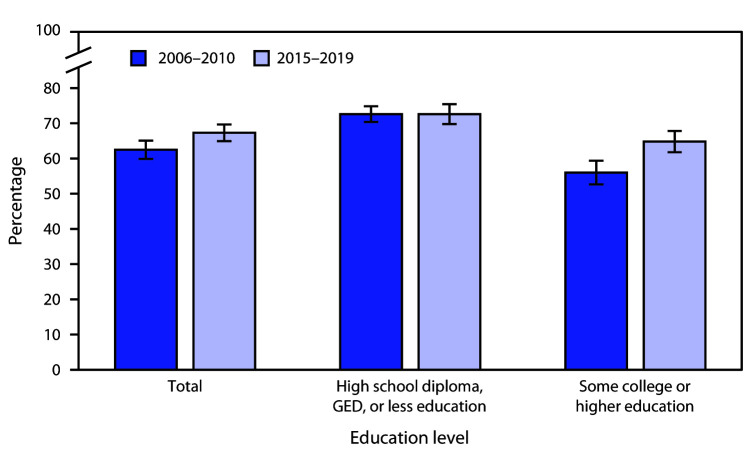
Among women aged 22–44 years, during 2015–2019, 67.3% had ever cohabited with an opposite-sex partner compared with 62.5% during 2006–2010. Among women with a high school diploma, GED, or less education, the percentages of those who had ever cohabited with an opposite-sex partner were similar (72.6%) across the two periods; the percentage of women with some college or higher education who had ever cohabited was higher for 2015–2019 (64.8%) than for 2006–2010 (56.0%). In both periods, women with a high school diploma, GED, or less education were more likely to have ever cohabited with an opposite-sex partner than were women with some college or higher education.

**Source:** National Survey of Family Growth, 2006–2010 and 2015–2019. https://www.cdc.gov/nchs/nsfg/index.htm.

